# Implications of Nonstationary Effect on Geographically Weighted Total Least Squares Regression for PM_2.5_ Estimation

**DOI:** 10.3390/ijerph18137115

**Published:** 2021-07-02

**Authors:** Arezoo Mokhtari, Behnam Tashayo, Kaveh Deilami

**Affiliations:** 1 Department of Geomatics Engineering, Faculty of Civil Engineering and Transportation, University of Isfahan, Isfahan 8174673441, Iran; a.mokhtari@trn.ui.ac.ir; 2 Centre for Urban Research, School of Global, Urban and Social Studies, RMIT University, Melbourne, VIC 3001, Australia; kaveh.deilami@rmit.edu.au

**Keywords:** land use regression, PM_2.5_, weighted total least squares, geographically weighted regression, ordinary least squares

## Abstract

Land use regression (LUR) models are used for high-resolution air pollution assessment. These models use independent parameters based on an assumption that these parameters are accurate and invariable; however, they are observational parameters derived from measurements or modeling. Therefore, the parameters are commonly inaccurate, with nonstationary effects and variable characteristics. In this study, we propose a geographically weighted total least squares regression (GWTLSR) to model air pollution under various traffic, land use, and meteorological parameters. To improve performance, the proposed model considers the dependent and independent variables as observational parameters. The GWTLSR applies weighted total least squares in order to take into account the variable characteristics and inaccuracies of observational parameters. Moreover, the proposed model considers the nonstationary effects of parameters through geographically weighted regression (GWR). We examine the proposed model’s capabilities for predicting daily PM_2.5_ concentration in Isfahan, Iran. Isfahan is a city with severe air pollution that suffers from insufficient data for modeling air pollution with conventional LUR techniques. The advantages of the model features, including consideration of the variable characteristics and inaccuracies of predictors, are precisely evaluated by comparing the GWTLSR model with ordinary least squares (OLS) and GWR models. The $R^{2}$ values estimated by the GWTLSR model during the spring and autumn are 0.84 and 0.91, respectively. The corresponding average $R^{2}$ values estimated by the OLS model during the spring and autumn are 0.74 and 0.69, respectively, and the $R^{2}$ values estimated by the GWR model are 0.76 and 0.70, respectively. The results demonstrate that the proposed functional model efficiently described the physical nature of the relationships among air pollutants and independent variables.

## 1. Introduction

Urban population growth and industrial development have led to several adverse environmental impacts, including land use change and widespread land degradation [1,2]. These changes contribute to high pollutant concentrations and increased air pollution. Long-term exposure to pollution sources poses the highest risk to human health. Studies have investigated long-term pollution exposure, which can increase mortality rate even if other risk factors, such as smoking, are controlled [3,4]. According to the World Health Organization (WHO), 4% of global mortality can be attributed to air pollution [5]. Due to economic reasons, Asian countries are experiencing greater air pollution levels and higher mortality rates [6,7]. The WHO reported that approximately 4.2 million deaths were due to air pollution in 2016. Every year, worldwide, air pollution is estimated to cause about 16% of mortalities due to lung cancer, 17% due to heart disease, 25% due to brain stroke, and 26% due to respiratory diseases [8,9,10,11].

Due to their minute dimensions, PM_2.5_ particles can penetrate into various body tissues and cause high pathogenicity [12,13,14]. Urban traffic is the most significant contributor to increased PM_2.5_ pollution [15,16,17]; industries are another significant source of the increase in PM_2.5_ concentration. Therefore, populations in highly dense industrial cities are more vulnerable to air pollution-related diseases. For this reason, in such areas, high-resolution estimation of air pollution concentration has become an important issue.

Previous studies have used air pollution monitoring stations to determine levels of exposure. However, considering the variation in pollution concentrations in cities and the costs associated with a large number of monitoring stations, such methods are inefficient at an urban scale [18,19,20]. Alternatively, another group of studies employed portable monitoring devices to explore spatial variations in air pollution; however, these devices pose several limitations when used in large regions [21,22,23]. Due to the difficulties associated with direct measurements of pollutant concentrations, several methods, such as interpolation, dispersion, and LUR have been developed to generate high-resolution data [24,25,26,27]. LUR is a spatial modeling method introduced by Briggs et al. [24]. LUR-based methods entail lower computational costs and have broader applications as compared with dispersion models. Furthermore, they produce small-scale pollutant variations, and thus outperform the interpolation methods for estimating pollution concentration [28].

Models have been developed following the LUR method by considering the pollutant concentration at monitoring stations as the dependent variable and using independent variables such as traffic, land use, and meteorological parameters with the strongest correlations with pollutant concentration [24,29,30,31,32]. Among the various techniques for LUR-based air pollution modeling, there are nonlinear methods with different time scales (daily, monthly, or annual) that use a weighted support vector regression. Such methods consider the relationships among the dependent variable and the independent variables to be constant.

If spatial heterogeneity is present in the observations, an OLS regression yields inaccurate regression coefficients. As a result, studies have considered GWR and mixed-effect models to address this issue [33,34,35,36]. These methods consider the spatial heterogeneity in the observational data and apply a weight matrix to develop the model and to determine the coefficients. The weight matrix is based on the distances between the observation points and monitoring stations. In areas with spatial heterogeneity, the GWR method reveals more details than the OLS method, since the GWR model considers spatial variations at every location, thereby producing more reliable results. However, both of the GWR and OLS models consider the independent variables as accurate and invariable parameters [37].

Solving a regression problem starts by determining a functional model for evaluating the issue. Physical phenomena are described by various functional models specified by dependent and independent variables and their relationships [38]. The primary point when solving a LUR problem is the method used for calculating the functional model coefficients. Most studies have used standard linear regression techniques to develop models for estimating air pollution concentration. The least squares method is commonly used to determine linear regression models’ coefficients by minimizing the root squared error. The coefficient calculation in regression models is performed to improve the accuracy of estimation and modeling of dependent variables. Air pollution modeling commonly considers independent variables such as traffic, meteorological parameters, and land use, all of which are determined by measurement or modeling. Although measurement and modeling inaccuracies and variabilities associated with independent variables have an adverse effect on the estimation accuracy, land use regression models do not take into account the impact of measurement or modeling errors on the independent variables. 

In this study, we propose an integrated GWR and a weighted total least squares (WTLS) regression model to improve the existing relations and modeling accuracy. The WTLS method can take into account observational inaccuracies in LUR models. The proposed GWTLSR model introduces random errors into both the design matrix and the observation vector. Hence, the measurement inaccuracies are taken into account when calculating the independent variables. Furthermore, the method’s accuracy and reliability are improved by considering the spatial heterogeneity through GWR. To evaluate the GWTLSR method’s efficiency, a nonlinear weighted LUR model is developed to model the nonlinear relationships among the independent variables, including traffic, meteorological parameters, and land use, and the dependent variable PM_2.5_ for Isfahan, Iran.

## 2. Methods and Data

This section starts with an introduction to OLS regression. Next, the development of the OLS-based GWR is described. The capabilities of the WTLS method for estimating regression coefficients are subsequently discussed. Then, it presents how GWR and WTLS are integrated to develop an accurate and reliable model to describe the nonlinear relationship among the independent variables, including traffic, meteorological parameters, and land use on the one hand and PM_2.5_ concentration on the other hand.

### 2.1. Ordinary Least Squares Regression

The ordinary least squares method is a statistical technique for estimating the unknown parameters of a linear regression model. OLS minimizes an objective function ∅ representing the sum of squares of the differences between the observed values and the values obtained from the data-fitted model [39]. The objective function to be minimized in the OLS regression is as follows:

(1) $$\emptyset =e_{y}^{T}Q_{y}^{-1}e_{y}$$

where $e_{y}$ is the error matrix of the observation vector **y**, if the dimensions of the observation vector are m×1, then, the dimensions of $e_{y}$ will also be m×1 and $Q_{y}$ is the m×m covariance matrix of observations (observations weight matrix). In the OLS model, an equal weight of 1 is assigned to all the observations. Therefore, the covariance matrix in this model is an identity matrix that can be ignored. This model considers that the independent variables are invariable and error-free observations and uses a simple functional model (Equation (2)). The unknown coefficients are calculated based on the method of least squares according to Equation (3) as follows:

(2) $$y=Ax+e_{y}$$

(3) $$\hat{x}={\left(A^{T}A\right)}^{-1}A^{T}y$$

In Equation (3), **A** is the m×n design matrix and **x** is the n×1 vector of unknowns.

### 2.2. Geographically Weighted Regression

On the basis of the theory of OLS, the GWR method was developed by Fotheringham et al. (2003) to consider spatial heterogeneity. The GWR model is the same as a modified moving windows model. The difference is that the points within the windows of interest are weighted differently, i.e., proportional to the target points [39,40]. In other words, the GWR model provides a local estimation rather than a global estimation of the parameters. Therefore, GWR is one of the best modeling techniques for spatially heterogeneous phenomena. Its general form can be expressed as follows [35,40]:

(4) $$y_{i}=x_{0}\left(u_{i}.v_{i}\right)+\overset{m}{\underset{k=1}{\sum }}x_{k}\left(u_{i}.v_{i}\right)a_{ik}+\varepsilon_{i}$$

where $\left(u_{i}.v_{i}\right)$ represent the coordinates of the ith sample in the study space; $x_{0}\left(u_{i}.v_{i}\right)$ is the intercept of the linear data-fitted model at location *i*; $x_{1}\left(u_{i}.v_{i}\right)$ to $x_{k}\left(u_{i}.v_{i}\right)$ are the local regression coefficients of the 1st to the kth independent variables at location *I*; $y_{i}$ represents the dependent variable at location *i*; and $a_{ik}$ is *k*th independent variable at location *i* [40]. 

Since samples (observations) usually outnumber unknowns, the unknown coefficients should be estimated using the least squares method, which is given by:

(5) $$\hat{x}\left(u_{i}.v_{i}\right)={\left(A^{T}W\left(u_{i}.v_{i}\right)A\right)}^{-1}*A^{T}W\left(u_{i}.v_{i}\right)y$$

where $W\left(u_{i}.v_{i}\right)$ is an n×n diagonal matrix whose main diagonal entries represent the weights of the GWR model kernel at location *i* [40,41]. In this study, a fixed Gaussian-based kernel was used (Equation (6)) as follows:

(6) $$W_{k}\left(u_{i}.v_{i}\right)=exp\left(-\frac{{\left(d_{k}^{s}\left(u_{i}.v_{i}\right)\right)}^{2}}{b^{2}}\right)$$

where $W_{i}$ is the geographical weight of the *k*th observation at location *i*, *b* is bandwidth, and *d* is the distance between the location of the *k*th observation and the location of *i*.

### 2.3. GWTLSR

The regression coefficients in both of the GWR and OLS models are calculated by minimizing the sum of squared errors. These models use the standard least squares method. These models only consider errors in the observation vector, and the design matrix (A) is assumed to be accurate and error free. In air pollution modeling, PM_2.5_ concentration and also the values of independent variables are obtained by measurement or modeling. Hence, there are errors-in-variables (EIV). The ordinary least squares method does not yield accurate solutions for such problems. Weighted total least squares can be used to solve the previously mentioned problem, introduced by Markovsky et al. in 2006 [42]. Due to the widespread use of least squares methods in various scientific fields, different formulations have been proposed for the WTLS method. In this study, an OLS-based formulation for the total least squares [43] was applied to develop the land use regression model. 

Contrary to OLS, the WTLS model considers errors in the design matrix and the observation vector. Therefore, the total least squares method can improve modeling accuracy if random errors influence the coefficient matrix. In air pollution modeling, the design matrix entries are the independent variables that include modeled traffic variables, land use parameters derived from aerial images and field surveys, and meteorological parameters measured at synoptic stations. Each of these parameters has an independent measurement accuracy. As a result, WTLS enables taking the independent variables’ inaccuracies into account along with the dependent variable’s inaccuracies and can be used more efficiently to determine the model coefficients.

Similar to OLS, the total least squares method minimizes the sum of squared differences between observational values and the data-fitted model’s values. The difference is that it must also simultaneously minimize design matrix errors along with minimizing the observation vector errors. To solve the WTLS problem, this study used Lagrange multipliers to minimize the objective function ∅ given by the following:

(7) $$\emptyset =e_{y}^{T}Q_{y}^{-1}e_{y}+e_{A}^{T}Q_{A}^{-1}e_{A}+2\lambda^{T}\left(y-Ax-e_{y}+\left(x^{T}⊗l_{m}\right)e_{A}\right)$$

where $l_{m}$ is an m×m identity matrix, **λ** represents the m×1 vector of Lagrange multipliers, $e_{A}$ is an mn×1 vector of design matrix errors, and x indicates the n×1 vector of unknown.

Taking the first derivative of Equation (7) with respect to each of the main values, one obtains the following four equations:

(8) $$\frac{1}{2}\frac{\partial \emptyset }{\partial e_{y}^{T}}=Q_{y}^{-1}{\tilde{e}}_{y}-\hat{\lambda }=0$$

(9) $$\frac{1}{2}\frac{\partial \emptyset }{\partial e_{A}^{T}}=Q_{A}^{-1}{\tilde{e}}_{A}+\left({\hat{x}}^{T}⊗l_{m}\right)\hat{\lambda }=0$$

(10) $$\frac{1}{2}\frac{\partial \emptyset }{\partial \lambda^{T}}=y-A\hat{x}-{\tilde{e}}_{y}+\left({\hat{x}}^{T}\;l_{m}\right){\tilde{e}}_{A}=0$$

(11) $$\frac{1}{2}\frac{\partial \emptyset }{\partial x^{T}}=-\left(A^{T}\hat{\lambda }-{\tilde{E}}_{A}^{T}\hat{\lambda }\right)=0$$

By simultaneously solving the set of Equations (8)–(11), the $\hat{\lambda }$, ${\tilde{e}}_{A}$, $Q_{\tilde{y}}$, and $\hat{x}$ values are obtained as follows:

(12) $$\hat{\lambda }={\left(Q_{y}+\left({\hat{x}}^{T}⊗l_{m}\right)Q_{A}\left(\hat{x}⊗l_{m}\right)\right)}^{-1}\left(y-A\hat{x}\right)$$

(13) $${\tilde{e}}_{A}=vec{\tilde{E}}_{A}=-Q_{A}\left({\hat{x}}^{T}⊗l_{m}\right)\hat{\lambda }$$

(14) $$Q_{\tilde{y}}=Q_{y}+\left({\hat{x}}^{T}⊗I_{m}\right)Q_{A}\left(\hat{x}⊗I_{m}\right)$$

(15) $$\hat{x}={\left({\left(A-{\tilde{E}}_{A}\right)}^{T}Q_{\tilde{y}}^{-1}\left(A-{\tilde{E}}_{A}\right)\right)}^{-1}{\left(A-{\tilde{E}}_{A}\right)}^{T}Q_{\tilde{y}}^{-1}\left(y-{\tilde{E}}_{A}x\right)$$

The vec operator in Equation (13) reshapes the *mn* vector to an *m* × *n* matrix. The relations for total least squares and ordinary least squares are similar in form, as indicated by Equation (15). In Equation (15), $\tilde{A}=A-{\tilde{E}}_{A}$ is the design matrix, $Q_{\tilde{y}}=Q_{y}+\left({\hat{x}}^{T}⊗l_{m}\right)Q_{A}\left(\hat{x}⊗l_{m}\right)$ represents the covariance matrix, and $\tilde{y}=y-{\tilde{E}}_{A}x$ is the observation vector. Here, the aim is to calculate $\hat{x}$ to determine the modeling coefficients. Considering the general forms of these equations, the equation can be solved using an iterative mechanism and determining the unknowns’ initial values by employing OLS.

A hybrid method that consolidates WTLS and GWR is proposed to consider spatial heterogeneity, variable characteristics, and inaccuracies in air pollution modeling variables. This model applies WTLS to determine the GWR coefficients. The general formula used in the model has the following form:

(16) $$y=\left(A-E_{A}\right)x+e_{y}$$

where y is the observation matrix consisting of the dependent variables, **A** is the design matrix carrying the values of the independent variables as well as a column of coefficient 1 representing the intercept of the model, **x** represents the coefficient values of the independent variable, and $E_{A}$ is the random errors of the design matrix A, which is defined as follows:

(17) $$A=\left[\begin{array}{ccccc} a_{11} & \cdots & a_{1k} & & 1\\ \vdots & \ddots & \vdots & & \vdots \\ a_{m1} & \cdots & a_{mk} & & 1 \end{array}\right]$$

where $a_{ik}$ is the value of the kth independent variable in location *i*.

(18) $$y=\left[\begin{array}{c} y_{1}\\ \vdots \\ y_{m} \end{array}\right]$$

where $y_{i}$ is the value of the dependent variable in location *i*.

(19) $$x=\left[\begin{array}{c} x_{1}\left(u_{i}.v_{i}\right)\\ x_{2}\left(u_{i}.v_{i}\right)\\ \vdots \\ x_{k}\left(u_{i}.v\right)\\ x_{0}\left(u_{i}.v_{i}\right) \end{array}\right]$$

where $x_{k}\left(u_{i}.v_{i}\right)$ is the coefficient of the kth variable and $x_{0}\left(u_{i}.v_{i}\right)$ is the intercept, located at $\left(u_{i}.v_{i}\right)$.

(20) $$E_{A}=-vec^{-1}\left(Q_{A}\left(\hat{x}⊗I_{m}\right)Q_{y}^{-1}\hat{e}\right),\;\;\;\hat{e}=y-Ax$$

where the operator, $vec^{-1}$, converts an mn×1 vector to an m×n matrix; $Q_{y}^{-1}$ is the matrix of the measured weights in the GWR model; and $Q_{A}$ is the weight matrix of the independent variables. $Q_{A}$ and $Q_{y}$ are defined as follows:

(21) $$Q_{A}=\left[\begin{array}{ccccccc} \delta_{a_{11}}^{2} & 0 & 0 & & 0 & \cdots & 0\\ 0 & \ddots & 0 & & 0 & \cdots & 0\\ 0 & 0 & \delta_{a_{mk}}^{2} & & 0 & \cdots & 0\\ 0 & 0 & 0 & & 0 & \cdots & 0\\ \vdots & \vdots & \vdots & & \vdots & \ddots & \vdots \\ 0 & 0 & 0 & & 0 & \cdots & 0 \end{array}\right]$$

(22) $$Q_{y}=W^{-1}={\left[\begin{array}{ccc} W_{1}\left(u_{i}.v_{i}\right) & 0 & 0\\ 0 & \ddots & 0\\ 0 & \cdots & W_{m}\left(u_{i}.v_{i}\right) \end{array}\right]}^{-1}$$

where $\delta_{a_{ik}}^{2}$ represents the measurement accuracy (variances) associated with the kth independent variable in the ith sample, and the values $W_{m}\left(u_{i}.v_{i}\right)$ in Equation (22) are the measured weights in GWR based on the neighboring distance.

**Figure 1:**
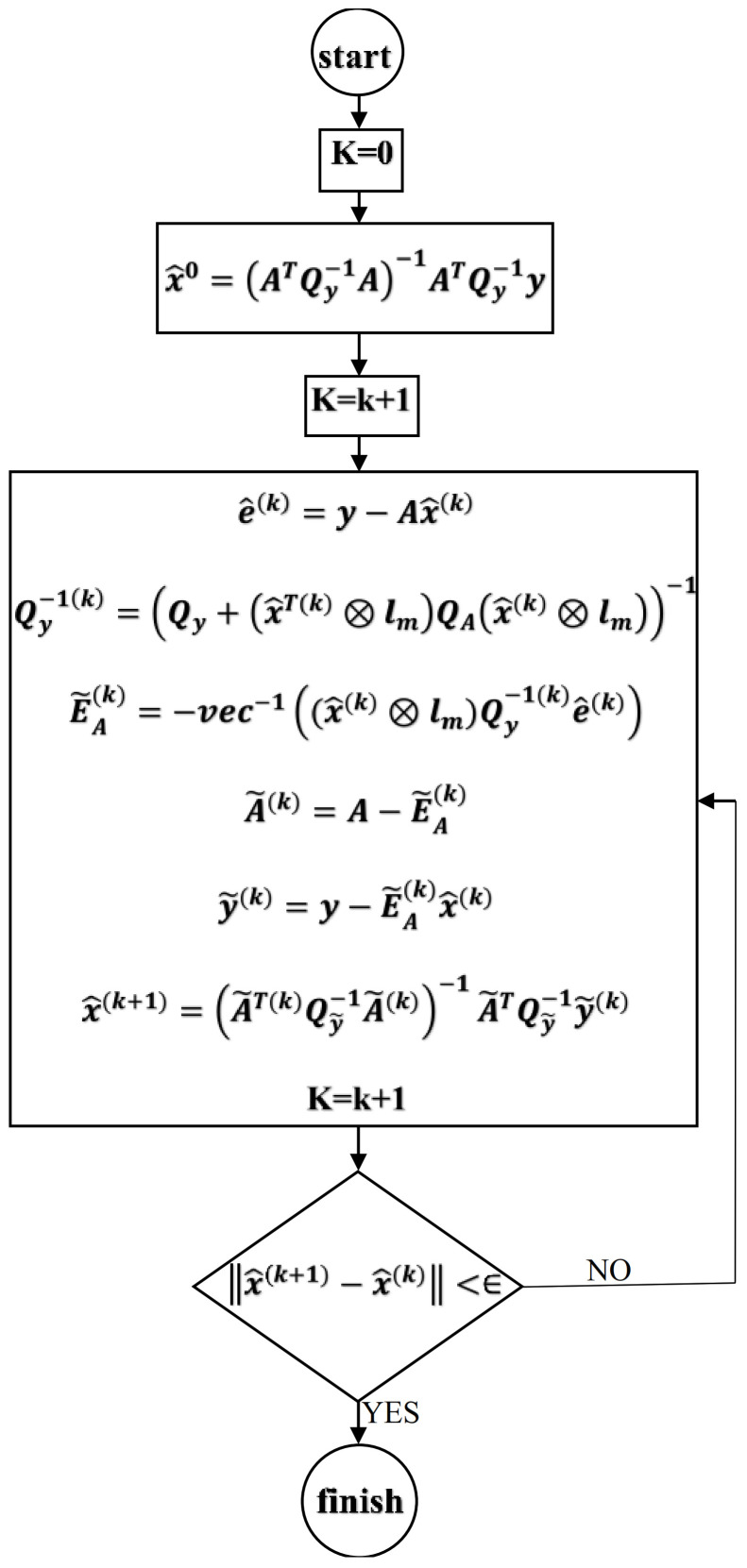
Schematic algorithm of the hybrid model.

Once the covariance matrices are determined, Equation (15) and total least squares are used to calculate the model coefficients following an iterative process (Figure 1).

### 2.4. Comparison of Regression Models

The Akaike information criterion ($AIC_{c}$) coefficient, the coefficient of determination, the root mean squared error (RMSE), and the mean absolute error (MAE) were employed to assess the regression models’ performance. $AIC_{c}$ is calculated as follows:

(23) $$AIC_{c}\left(b\right)=2nln\left(\hat{\sigma }\right)+nln\left(2\pi \right)+n\left\{\frac{n+tr\left(S\right)}{n-2-tr\left(S\right)}\right\}$$

where n, b, and $\hat{\sigma }$ are the sample size, bandwidth, and standard deviation of the model, respectively. Each row of the matrix S that contains independent variables is determined according to Equation (24) [44]:

(24) $$r_{i}=A\left(i,:\right)\;{\left(A^{T}W_{i}A\right)}^{-1}A^{T}W_{i}$$

(25) $$R^{2}=1-\frac{\sum_{i=1}^{n}{\left({\hat{y}}_{i}-y_{i}\right)}^{2}}{\sum_{i=1}^{n}{\left(y_{i}-\bar{y}\right)}^{2}}$$

(26) $$\mathrm{RMSE}=\sqrt{\frac{1}{n}\overset{n}{\underset{i=1}{\sum }}{\left({\hat{y}}_{i}-y_{i}\right)}^{2}}$$

(27) $$\mathrm{MAE}=\frac{\sum_{i=1}^{n}\left|y_{i}-{\hat{y}}_{i}\right|}{n}$$

In Equations (25)–(27), *n* is the sample size, $y_{i}$ is *i*-th station observed to be dependent variable, $\bar{y}$ is the mean values of the independent variable, and ${\hat{y}}_{i}$ is the estimated value of the dependent variable.

Moreover, two statistical metrics, the probability of false alarm (POF) and the probability of detection (POD) [45,46], were applied to evaluate the regression model’s performance for estimating PM_2.5_ concentration.

(28) $$\mathrm{POD}=\frac{a}{a+b}$$

(29) $$\mathrm{POF}=\frac{c}{a+c}$$

where *a* is the number of events in which both observed and estimated PM_2.5_ concentrations are higher than the 90th percentile (successful predictions); *b* is the number of events in which the estimated PM_2.5_ concentration is lower than the 90th percentile, whereas the observed PM_2.5_ concentration is higher than the 90th percentile (unsuccessful predictions); *c* is the number of events in which the estimated PM_2.5_ concentration is higher than the 90th percentile, whereas the observed PM_2.5_ concentration is lower than the 90th percentile (wrong alarms). In a perfect prediction, POD equals one, and POF equals zero. 

### 2.5. Study Area and Data Preparation

**Figure 2:**
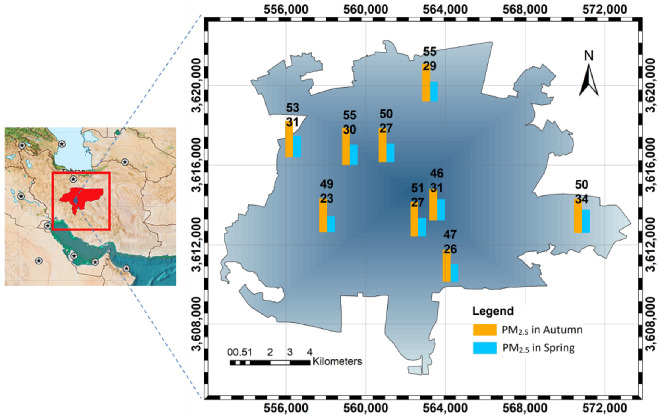
Distribution of air pollution monitoring stations in zone 39N of the universal Transverse Mercator (UTM) coordinate system and seasonal PM_2.5_ concentrations.

With a population of more than 2.1 million, Isfahan is one of the most populous and polluted industrial cities in central Iran (Figure 2). It is home to many industrial units as one of Iran’s industrial hubs. According to previous studies, traffic, residential land use, and nonresidential land use are responsible for 76%, 11%, and 13% of emissions in Isfahan on a daily basis, respectively [47]. Therefore, in this study, the LUR model was developed based on these predictors to estimate the PM_2.5_ pollutant concentration. 

The study area included nine monitoring stations and the target pollutant concentrations were measured in one-hour intervals from 2017 to 2019 during the spring and autumn. The seasons are considered according to calendar seasons. The calculated average daily concentrations were used for modeling purposes. After checking the quality of the measured data at each station and removing the biased samples, on average, each station contained 260 and 240 measurements in spring and autumn, respectively (Figure 2).

The independent variables used in this study were traffic, land use, and meteorological parameters, as they have the most significant effect on PM_2.5_ concentration in Isfahan [48,49]. These variables are described as follows.

1. The meteorological parameters included temperature, humidity, precipitation, pressure, and wind speed measured by the Isfahan Weather Forecast Organization at the synoptic stations. The measurements were taken in three-hour intervals during the spring and autumn from 2017 to 2019. The daily average of the measured value was considered to be an independent variable at each station.
2. The traffic data obtained from the Isfahan Municipality included traffic volume from a four-stage transportation model. First, the hourly traffic counts were determined for sample days during the spring and autumn from 2017 to 2019. Second, they were extracted in buffers around the monitoring stations of 150, 300, 600, and 1200 m radius. Finally, the buffers with the highest correlation were eventually kept in the model.
3. The land use data were derived from a 2019 map (scale = 1:2000). The original land use classes were reclassified into residential and non-residential classes. Subsequently, buffers of 100, 200, and 500 m radius were used to estimate the relevant independent variables.

**Figure 3:**
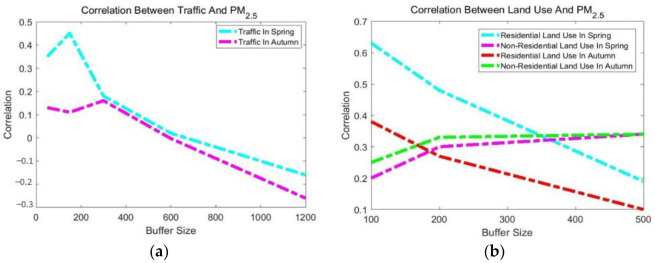
Correlation coefficients between PM_2.5_ concentration and independent variables ((**a**) traffic and (**b**) land use) for various buffers in spring and autumn.

In order to improve modeling accuracy, the significant independent variables were selected from all variables using a two-stage process. The first stage used Pearson’s correlation coefficient to choose the variables bearing the strongest correlation to the independent variables (Figure 3). In the second stage, various combinations of the selected variables were applied to develop OLS-based models. The variables with *p*-values more than 0.05 were ignored. Finally, the variables having the most significant effect on the $R^{2}$ were selected to develop the OLS, GWR, and GWTLSR models.

Figure 3 shows the correlation coefficients between the independent variables and PM_2.5_ concentration in the spring and autumn. On the one hand, the results showed that, in the autumn, the following variables had the highest correlation with PM_2.5_ concentration: the 1200 m buffer traffic volume with a negative relationship and residential and non-residential land use in the 100 m and 500 m buffers with a direct relationship. Among the meteorological parameters, temperature with a negative relationship and pressure directly bore the highest correlations with PM_2.5_ concentration. In the spring, on the other hand, the variables bearing the highest correlation with PM_2.5_ concentration were residential and non-residential land use in the 100 m and 500 m buffers and traffic in a 150 m buffer, all with a direct effect. In addition, temperature and pressure were among the variables bearing the highest correlation with PM_2.5_ concentration with positive and negative relationships. The temperature relationship with PM_2.5_ was considerably more significant in autumn than spring due to lower temperatures and thermal inversion. 

## 3. Results and Discussion

**Figure 4:**
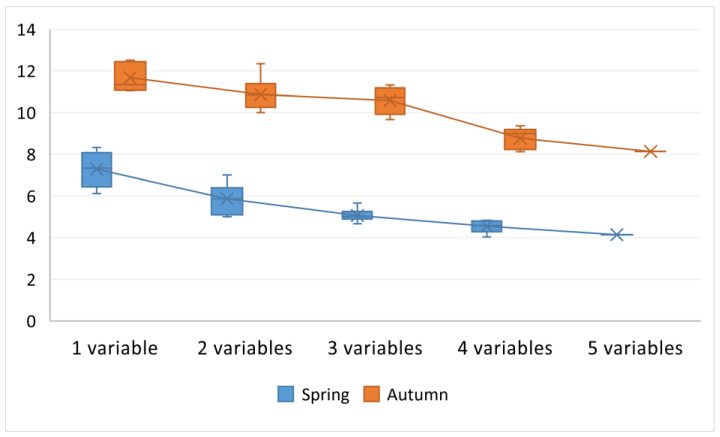
Distribution of root mean square error (RMSE) in autumn and spring with respect to the number of independent variables.

Figure 4 presents the distribution of RMSE in the OLS model using combinations of one to five independent variables, which are most closely correlated with PM_2.5_. As shown in Figure 4, once the number of independent variables increased from one to five, the value of RMSE decreased accordingly. Therefore, a combination of five independent variables was eventually used in the final model. The selected independent variables applied in the spring were traffic in the 150 m buffer, residential and non-residential land use in the 100 m and 500 m buffers, respectively, temperature, and pressure. The selected independent variables applied in the autumn were traffic in the 300 m buffer, residential and non-residential land use both in the 200 m buffers, temperature, and pressure.

In order to examine the selected independent variables, the *p*-value and variance inflation factor (VIF) index of the selected variables have been presented (Table 1). The closer the VIF index values of each independent variable are to one, the better the variable is selected in the model. There are no multiple alignments among the chosen variables.

Table 2 shows the results of the three models for the spring and autumn. Model performance was evaluated by cross-validation. Given the number of stations in the study area, the models were validated by the leave-one-out cross-validation method. One station was left out from the observations at each stage in this method, and the models were developed using the remaining stations. According to the number of monitoring stations in the study area, the data used were divided into nine sections. At each stage, observations of one station were considered to be testing data and the remaining stations were considered to be training data. This process was repeated nine times and, in each step, observations of one station were considered to be test data. The modeling accuracy was determined at the end of the process based on the mean accuracy of all stages. The mean values of RMSE and MAE for the test and training data sets and $AIC_{c}$ of the models are shown in Table 2. The results demonstrated the superior performance of the GWTLSR method as compared with the other modeling techniques. A reduction in the $AIC_{c}$ coefficient accompanied the improved performance as compared with the other models. This reduction indicated the high computational efficiency of the proposed model. The accuracy was improved without any substantial increase in computational complexity. The absence of a significant difference between the validation results for the two seasons demonstrated the proposed model’s stability.

**Figure 5:**
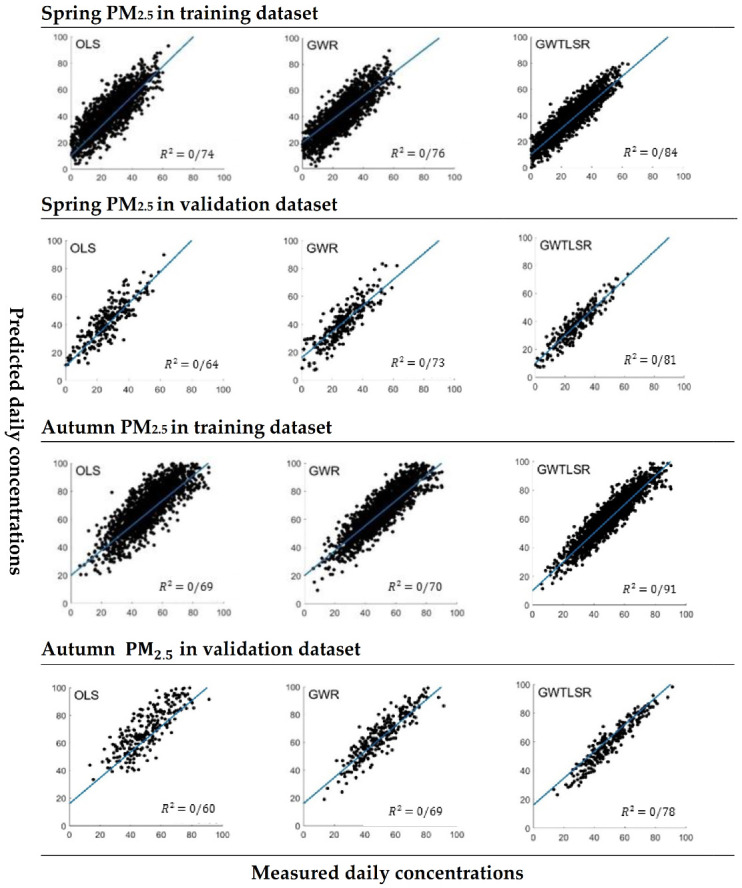
Scatter plots of estimated (y axis) against observed (x axis) for PM_2.5_ (μg/m^3^) for the test and training data sets.

Figure 5 shows that the integrated GWTLSR model explains 0.82 and 0.92 of the total variances of PM_2.5_ in the spring and autumn, respectively. While the corresponding values were, respectively, 0.74 and 0.69 for the OLS model and 0.7 and 0.76 for the GWR model. The higher $R^{2}$. coefficient of the proposed model showed that it was superior in accuracy and covered the dependent variable distribution more broadly. The results presented in Table 1 and Figure 5 show that considering inaccuracy, varying characteristics, and nonstationary effects of independent variables simultaneously led to performance improvement of the GWTLSR model as compared with conventional models used in previous research.

In order to evaluate the significance of the accuracy difference in the studied models, one-way analysis of variance (ANOVA) with a confidence interval of 0.95% was used. The results of the analysis show the significant difference in accuracy among the studied models.

**Figure 6:**
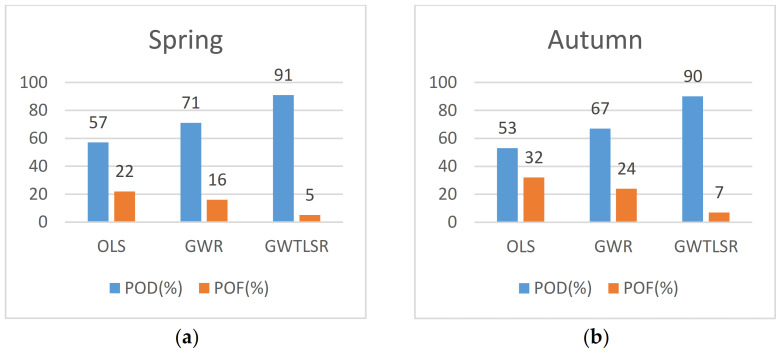
The probability of detection (POD (%)) and probability of false alarm (POF (%)) for estimation of PM_2.5_ in (**a**) spring and (**b**) autumn.

Figure 6 shows the average POD (%) values of GWTLSR for estimating PM_2.5_ concentration which was higher than the other models, in addition, the average GWTLSR POF (%) values were lower than the two other models for estimating PM2.5 concentration.

**Figure 7:**
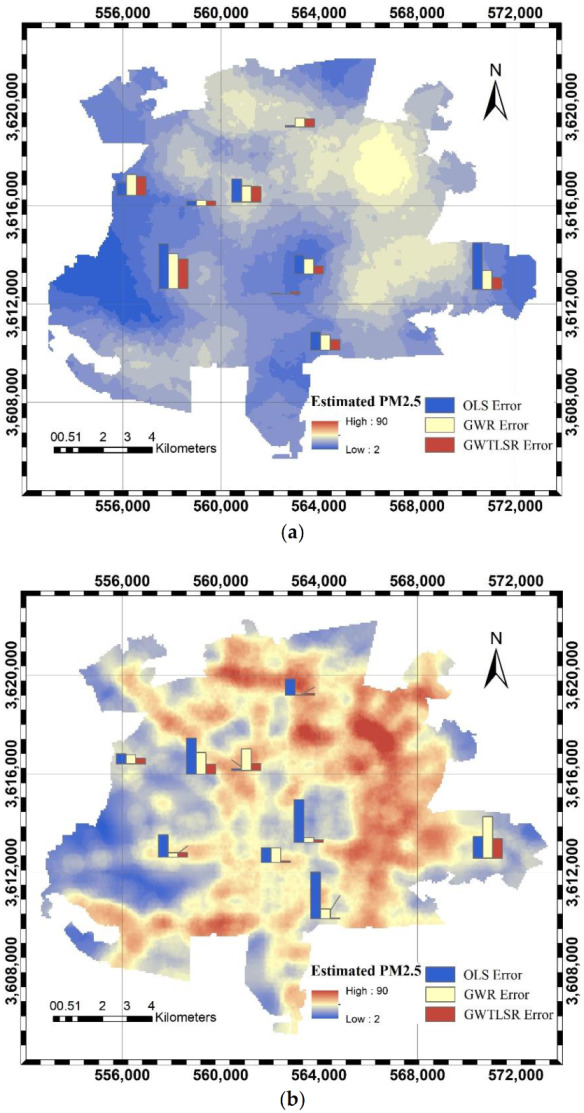
Estimated PM_2.5_ concentrations in (**a**) spring and (**b**) autumn, using the GWTLSR model and the models’ error at monitoring stations.

Figure 7 presents the PM2.5 concentrations obtained from the GWTLSR model for the spring and autumn. Moreover, it shows the differences between the estimated and observed PM2.5 concentrations in the spring and autumn at monitoring stations for the three modeling methods. The maximum differences obtained by the integrated model in the spring and autumn are 2.23 and 1.74, respectively. The corresponding differences are, respectively, 3.54 and 4.20 for the OLS model and 2.63 and 3.74 for the GWR model. In the OLS and GWR models, the differences between the estimated and observed concentrations increase as the pollutant concentration increased. However, the corresponding difference in the proposed model is not significant. Thus, it can be concluded that the performance of the proposed model is less affected by the mean and variance of the dependent parameters.

As shown in Figure 7, the Moran’s I Index was applied to examine the spatial autocorrelation of the models’ errors. Table 3 presents the Moran’s I Index values of the proposed model as −0.10 and −0.12, with *p*-values equal to 0.89 and 0.98 for the spring and autumn, respectively. Thus, the distribution of errors in the GWTLSR model is random. It can be concluded that the errors in the GWTLSR are not dependent on the monitoring station’s location due to the consideration of variable characteristics and nonstationary effects of parameters.

## 4. Conclusions

This study proposed a geographically weighted total least squares regression (GWTLSR) model for high-resolution mapping of air pollution. The proposed model simultaneously considered the independent and dependent variables to be observational parameters. It could also consider the nonstationary effects of the parameters affecting pollutant concentration. These model features enabled effective air pollution modeling through available parameters with spatial heterogeneity and different measurement accuracy levels.

In the development of the proposed model, the WTLS was applied to calculate the GWR coefficients. Random errors were introduced to both the design matrix and the observation vector by using the WTLS method. As a result, the GWTLSR model estimates the results by considering measurement inaccuracies of the independent variables. Furthermore, spatial heterogeneity is taken into account in the proposed model by using GWR. 

To assess the GWTLSR model’s performance, a nonlinear weighted LUR model was developed to model the nonlinear relationships among traffic, meteorological parameters, and land use as the independent variables, and PM_2.5_ as the dependent variable for spring and autumn in Isfahan, Iran. The proposed integrated model’s benefits were investigated by comparing it with two conventional LUR models, namely OLS and GWR. These conventional models consider the independent variables to be invariable and accurate. As a result, the covariance matrix of observations only includes the covariance of the dependent variable. In contrast, the covariance matrix in the GWTLSR model contains both dependent and independent variables’ values. The results show that despite the insignificant differences between the performances of the OLS and GWR models, the GWTLSR model’s accuracy increased significantly. In addition, comparing the results for the spring and autumn demonstrated that the variables’ values and variance had less influence on the proposed model’s performance than that of the other two. In conclusion, although all these methods are developed based on the OLS theory, the GWTLSR model is more compatible with the variables’ nature. Therefore, the proposed model can significantly contribute to enhance air pollution modeling.

## Figures and Tables

**Table 1:** Comparison of *p*-value and variance inflation factor (VIF) values in selected independent variables.

Season	Variables	*p*-Value	VIF
Spring	Temperature	$4\times 10^{-3}$	1.28
	Pressure	$3\times 10^{-5}$	$3\times 10^{-4}$
	Traffic	$8\times 10^{-6}$	1.04
	Residential land use	$7\times 10^{-7}$	1.004
	Non-residential land use	$8\times 10^{-8}$	1.012
Autumn	Temperature	$2.5\times 10^{-3}$	1.007
	Pressure	$2\times 10^{-9}$	$6\times 10^{-4}$
	Traffic	$5\times 10^{-9}$	1.06
	Residential land use	$5\times 10^{-3}$	1.04
	Non-residential land use	$4\times 10^{-3}$	1.01

**Table 2:** Results of the three models in spring and autumn for test and training datasets.

Season	Model	${RMSE}_{Test}$	${RMSE}_{Train}$	${MAE}_{Test}$	${MAE}_{Train}$	${AIC}_{c}$
Spring	OLS	5.19	4.96	4.43	4.15	777.8
	GWR	5.02	4.66	4.11	3.85	607.9
	GWTLSR	4.26	3.83	3.55	3.14	574.5
Autumn	OLS	8.66	8.36	6.81	6.55	909.2
	GWR	8.40	8.04	6.62	6.42	715.2
	GWTLSR	7.12	4.34	5.26	3.51	596.5

Note: MAE, mean absolute error; RMSE, root mean square error; $AIC_{c},$ Akaike information criterion); OLS, ordinary least squares; GWR, geographically weighted regression; GWTLSR, geographically weighted total least squares.

**Table 3:** Spatial autocorrelation (Moran’s I) of the model’s error.

Season	Model	Moran’s I	z-Score	*p*-Value	Pattern
Spring	OLS	0.19	2.03	0.04	Clustered
	GWR	−0.17	−0.35	0.72	Almost random
	GWTLSR	−0.10	0.13	0.89	Random
Autumn	OLS	0.17	2.05	0.03	Clustered
	GWR	−0.18	−0.41	0.68	Almost random
	GWTLSR	−0.12	−0.02	0.98	Random

## Data Availability

The datasets used and analyzed during the current study are available from the corresponding author on reasonable request. The code used during the current study are available from the corresponding author on reasonable request.
